# Characteristics, management, and outcome of pediatric patients with post‐transplant lymphoproliferative disease—A 20 years' experience from Austria

**DOI:** 10.1002/cnr2.1375

**Published:** 2021-03-23

**Authors:** Anna Füreder, Gabriele Kropshofer, Martin Benesch, Michael Dworzak, Sabine Greil, Wolf‐Dietrich Huber, Holger Hubmann, Anita Lawitschka, Georg Mann, Ina Michel‐Behnke, Thomas Müller‐Sacherer, Herbert Pichler, Ingrid Simonitsch‐Klupp, Wolfgang Schwinger, Zsolt Szepfalusi, Roman Crazzolara, Andishe Attarbaschi, Austrian Society of Pediatric Hematology and Oncology

**Affiliations:** ^1^ Department of Pediatric Hematology and Oncology St. Anna Children's Hospital Vienna Austria; ^2^ Division of Pediatric Hematology and Oncology, Department of Pediatrics and Adolescent Medicine Medical University of Innsbruck Innsbruck Austria; ^3^ Division of Pediatric Hematology and Oncology, Department of Pediatrics and Adolescent Medicine Medical University of Graz Graz Austria; ^4^ Division of Pediatric Cardiology, Department of Pediatrics and Adolescent Medicine, Pediatric Heart Center Medical University of Vienna Vienna Austria; ^5^ Department of Pediatrics and Adolescent Medicine Medical University of Vienna Vienna Austria; ^6^ Division of General Pediatrics, Department of Pediatrics and Adolescent Medicine Medical University of Graz Graz Austria; ^7^ Department of Pathology Medical University of Vienna Vienna Austria; ^8^ Division of Pediatric Pulmonology, Allergy and Endocrinology, Department of Pediatrics and Adolescent Medicine, Comprehensive Center Pediatrics Medical University of Vienna Vienna Austria

**Keywords:** hematopoietic stem cell transplantation, outcome, post‐transplant lymphoproliferative disease, solid organ transplantation, treatment

## Abstract

**Background:**

Management of pediatric post‐transplantation lymphoproliferative disorder (PTLD) after hematopoietic stem cell (HSCT) and solid organ transplantation (SOT) is challenging.

**Aim:**

This study of 34 PTLD patients up to 19‐years old diagnosed in Austria from 2000 to 2018 aimed at assessing initial characteristics, therapy, response, and outcome as well as prognostic markers of this rare pediatric disease.

**Methods and results:**

A retrospective data analysis was performed. Types of allografts were kidney (n = 12), liver (n = 7), heart (n = 5), hematopoietic stem cells (n = 4), lungs (n = 2), multi‐visceral (n = 2), small intestine (n = 1), and vessels (n = 1). Eighteen/34 were classified as monomorphic PTLD, with DLBCL accounting for 15 cases. Polymorphic disease occurred in nine, and non‐destructive lesions in six cases. One patient had a non‐classifiable PTLD. Thirteen/34 patients are surviving event‐free in first remission (non‐destructive, n = 4/6; polymorphic, n = 4/9; monomorphic, n = 6/18). Fourteen/34 patients lacked complete response to first‐line therapy, of whom seven died. Four/34 patients relapsed, of whom two died. In 3/34 patients, death occurred as a first event. The 5‐year overall and event‐free survival rates were 64% ± 9% and 35% ± 9% for the whole cohort. Among all parameters analyzed, only malignant disease as the indication for transplantation had a significantly poor influence on survival.

**Conclusions:**

This study shows PTLD still to be a major cause of mortality following SOT or HSCT in children. A continued understanding of the molecular biology of the disease shall allow to decrease treatment intensity for lower risk patients and to identify patients who may benefit from newer therapy approaches to improve outcome and decrease morbidity.

**Abbreviations**
BM: bone marrow
B‐NHL: B‐cell non‐Hodgkin lymphoma
CCR: continuous complete remission
CHOP: cyclophosphamide, doxorubicin, vincristine, and prednisolone
CNS: central nervous system
CR: complete remission
CSF: cerebrospinal fluid
DLBCL: diffuse large B‐cell lymphoma
EBER: EBV‐encoded RNA
EBV: Epstein‐Barr virus
EFS: event‐free survival
EPOCH: etoposide, prednisolone, vincristine, cyclophosphamide, and doxorubicin
GIT: gastrointestinal tract
HLA: human leukocyte antigen
HSCT: hematopoietic stem cell transplantation
IPNHLSS: International Pediatric Non‐Hodgkin Lymphoma Staging System
LDH: lactate dehydrogenase
LPD: lymphoproliferative disease
m‐COMP: modified ‐ cyclophosphamide, vincristine, methotrexate, and prednisolone
NK‐cells: natural killer cells
OS: overall survival
PB: peripheral blood
PCR: polymerase chain reaction
PD: progressive disease
PH: plasmacytic hyperplasia
PR: partial remission
PTLD: post‐transplant lymphoproliferative disease
R‐CHOP: rituximab‐cyclophosphamide, doxorubicin, vincristine, and prednisolone
RIS: reduction of immunosuppression
SD: stable disease
SOT: solid organ transplantation
WHO: World Health Organization

## 1 INTRODUCTION

In transplantation medicine prevention and treatment of graft rejection is the primary aim of immunosuppressive medication. Besides this, iatrogenic immunosuppression is accompanied by serious side effects, such as an increased infection susceptibility and a higher risk of developing malignancies due to reduced tumor surveillance.^1^ The latter can result in post‐transplant lymphoproliferative disease (PTLD), which is a heterogeneous disorder ranging from benign hyperplasia to malignant lymphomas.^2^, ^3^, ^4^

In pediatric patients, PTLD represents the most frequent type of malignant diseases secondary to solid organ transplantation (SOT) or hematopoietic stem cell transplantation (HSCT), with the overall risk of developing malignancies being 45‐fold higher than in healthy individuals.^5^, ^6^, ^7^ Epstein‐Barr virus (EBV) infection is detected in most cases of pediatric PTLD.^8^, ^9^, ^10^, ^11^, ^12^, ^13^, ^14^, ^15^ Whereas >90% of the world's adult population already harbor EBV, the infestation rate is comparatively low in children.^16^, ^17^ Transplantation of grafts from EBV‐seropositive adults results in primary EBV infection of children, thereby explaining a 2‐ to 4‐fold higher risk of PTLD in children compared to adults.^5^, ^15^, ^18^, ^19^, ^20^ Reflecting the age‐dependent seroprevalence rates, EBV‐positivity occurs in only 50% of adult as compared to 85%‐90% of pediatric PTLD.^15^, ^16^, ^19^, ^21^, ^22^, ^23^ Notably, pediatric EBV‐negative PTLD typically develops late, that is, >1 year after transplantation.^4^, ^8^, ^11^, ^16^, ^24^

The World Health Organization (WHO) classification system differentiates between (i) non‐destructive, benign forms, showing polyclonal cell populations only, (ii) polymorphic subtypes, which can present with either polyclonal or monoclonal proliferation patterns, and (iii) monomorphic, monoclonal subtypes, which are genuine malignant lymphomas, mostly of B‐ and, less frequently, of T‐ or NK‐cell origin.^8^, ^9^

The incidence of lymphoproliferative diseases (LPD) following transplantations varies between 1% and 30%, depending on the presence of risk factors,^25^, ^26^ the most important of which are a mismatched EBV serostatus between recipient and donor,^8^, ^10^, ^11^, ^27^, ^28^ a high‐intensity immunosuppressive regimen,^3^, ^11^, ^12^, ^19^, ^29^ and the type of allograft. The highest risk for PTLD after SOT results from having an EBV‐positive organ donor and an EBV‐naïve recipient. For PTLD following HSCT, a converse serological EBV constellation of donor and recipient is an established risk factor. Transplantation of intestine or lungs poses the highest risk for PTLD (20%),^12^, ^26^, ^30^, ^31^, ^32^ followed by cardiac or liver (2%‐10%),^29^, ^33^, ^34^ and kidney transplantations (1%‐4%).^5^, ^19^, ^29^, ^35^, ^36^ HSCT shows an incidence of 1%‐8%, likewise depending on a range of risk factors, such as transplantation of T‐cell depleted bone marrow (BM) or HLA‐mismatched grafts from unrelated donors, as well as the use of anti‐thymocyte globulin.^37^, ^38^, ^39^, ^40^ Concerning the latency period after transplantation, LPDs can be grouped into early‐ and late‐onset diseases, referring to cases within and later than the first year after transplantation.^25^

Herein, we describe 34 SOT and HSCT patients with PTLD diagnosed in Austria within a period of nearly 20 years.

## 2 PATIENTS AND METHODS

### 2.1 Patients

Patients had to fulfill three inclusion criteria to be enrolled into this retrospective study: (i) diagnosis of PTLD as established by a reference pathologist according to the WHO classification valid at the time of diagnosis, (ii) diagnosis and treatment of PTLD between 2000 and 2018, and (iii) treatment of PTLD carried out at one of the four participating Austrian centers offering performance and aftercare of SOT and/or HSCT. Thirty‐four patients aged <19 years meeting the inclusion criteria were identified and, hence, included in our study.

## 3 METHODS

Classification of PTLD was based on histopathology according to the contemporary WHO classification system.^41^, ^42^ EBV‐association was defined by a positive in‐situ hybridization test of EBV‐encoded RNA (EBER) in the tissue(s) analyzed. Patient data included demographics and information on disease, treatment, response, and outcome. Serum analysis was performed to detect EBV in peripheral blood. In dependence of the laboratory, to which the samples were submitted, the threshold for EBV positivity was either ≥5 or ≥ 100 copies/ml. Tumor stage was retrospectively defined according to the International Pediatric Non‐Hodgkin Lymphoma Staging System (IPNHLSS) whenever possible.^43^, ^44^ Evaluation of response was documented at the end of each therapy line (complete remission, CR; partial remission, PR; stable disease, SD; progressive disease, PD). Accordingly, two subgroups were defined: (i) patients with complete response, including patients with CR at the end of a respective therapy line, and (ii) patients who lacked complete response, including all patients with PR, SD, and PD. Due to the lack of standardized imaging procedures used throughout the therapy and the retrospective nature of our study, our data on response were based on the results documented by the local treating physicians. Fatalities were classified as either (i) PTLD‐related, (ii) therapy of PTLD‐related, or (iii) PTLD‐unrelated deaths.

All patients were treated after informed consent from the patient, patient's parents or legal guardians had been obtained.

### 3.1 Statistical analysis

Event‐free survival (EFS) was calculated from the date of diagnosis to the date of first event. Events considered were lacking complete response at the end of a therapy line, relapse, or death, whichever occurred first. Overall survival (OS) was defined as the time from diagnosis to death from any cause or the date of last follow‐up. EFS and OS were estimated according to the Kaplan‐Meier method; differences between groups were evaluated with the log‐rank test. *P*‐values ≤0.05 were considered statistically significant.

## 4 RESULTS

### 4.1 Initial characteristics

Demographics, histopathological results and EBV‐status are shown in Tables 1 and 2. Among the 34 PTLD patients analyzed, median age was 8.78 years and the male‐to‐female ratio 1:1. Types of allografts were kidney (n = 12), liver (n = 7), heart (n = 5), hematopoietic stem cells (n = 4), lung (n = 2), multi‐visceral (n = 2), small intestine (n = 1), and vessels (n = 1). All patients received one or more immunosuppressive drugs at the time of PTLD onset with 24 receiving ≥2 immunosuppressive drugs.

**TABLE 1:** Clinical and laboratory characteristics of the 34 patients with PTLD according to the histological subtype

	Non‐destructive PTLD	Polymorphic PTLD	Monomorphic PTLD	Not classifiable PTLD	∑
*No. of patients*	6 (18%)	9 (26%)	18 (53%)	1 (3%)	34
*Gender*					
female	2 (33%)	5 (56%)	10 (56%)	0	17 (50%)
male	4 (67%)	4 (44%)	8 (44%)	1	17 (50%)
*Condition leading to Tx*					
malignant	0	0	3 (17%)	0	3 (9%)
non‐malignant, congenital	4 (67%)	5 (56%)	13 (72%)	0	22 (64%)
non‐malignant, acquired	2 (33%)	4 (44%)	2 (11%)	1	9 (27%)
*Organ Tx*					
liver^a^	3 (50%)	1 (11%)	3 (17%)	0	7 (21%)
heart	0	3 (33%)	2 (11%)	0	5 (15%)
lung	0	0	2 (11%)	0	2 (6%)
kidney	2 (33%)	3 (33%)	7 (39%)	0	12 (35%)
HSCT	0	0	3 (17%)	1	4 (12%)
other	1 (17%)	2 (22%)	1 (5%)	0	4 (12%)
*Time from Tx to PTLD*					
median (years)	1.23	1.04	0.63	/	0.65
range (years)	0.35‐2.56	0.19‐12.56	0.06‐11.68	/	0.06‐12.56
<1 year	3 (50%)	4 (44%)	11 (61%)	1	19 (56%)
≥1 year	3 (50%)	5 (56%)	7 (39%)	0	15 (44%)
*Age at PTLD onset*					
median	3.04	5.1	11.69	/	8.78
range	0.88‐16.63	2.52‐16.45	3.07‐21.81	/	0.88‐21.81
<10 years	4 (67%)	6 (67%)	9 (50%)	0	19 (56%)
≥10 years	2 (33%)	3 (33%)	9 (50%)	1	15 (44%)
*Immunsuppression at onset of PTLD*					
tacrolimus	6 (100%)	8 (89%)	12 (67%)	0	26 (77%)
prednisolone	3 (50%)	4 (44%)	11 (61%)	0	18 (53%)
mycophenolate mofetil	2 (33%)	5 (56%)	9 (50%)	0	16 (47%)
cyclosporin A	0	1 (11%)	5 (28%)	1	7 (21%)
other	1 (17%)	2 (22%)	2 (11%)	0	5 (15%)
1 drug	3 (50%)	1 (11%)	5 (28%)	1	10 (29%)
≥2 drugs	3 (50%)	8 (89%)	13 (72%)	0	24 (71%)
*B‐Symptoms*	3 (50%)	5 (56%)	4 (22%)	1	13 (38%)
*Pre‐therapeutic LDH level*					
≥500 U/L	4 (67%)	7 (78%)	13 (72%)	1	25 (74%)
<500 U/L	2 (33%)	2 (22%)	5 (28%)	0	9 (26%)

Abbreviations: No., number; Tx, transplantation; LDH; lactate dehydrogenase; PTLD, post‐transplant lymphoproliferative disease.

^a^ There was one patient included who developed the PTLD after liver transplantation, but had also undergone a previous kidney transplantation.

**TABLE 2:** Immunohistochemistry, EBV‐status, and site/stage of disease of the 34 patients with PTLD according to the histological subtype

	Non‐destructive PTLD	Polymorphic PTLD	Monomorphic PTLD	Not classifiable PTLD	∑
*No. of patients*	6 (18%)	9 (27%)	18 (53%)	1 (3%)	34
*CD20‐expression*					
positive	4 (67%)	8 (89%)	15 (83%)	1	28 (82%)
negative	2 (33%)	1 (11%)	3 (17%)	0	6 (18%)
*EBER status of samples*					
EBER‐positive	4 (67%)	9 (100%)	16 (89%)	1	30 (88%)
EBER‐negative	2 (33%)	0	2 (11%)	0	4 (12%)
*EBV‐PCR results of PB*					
positive	5 (83%)	9 (100%)	15 (83%)	1	30 (88%)
negative	1 (17%)	0	1 (6%)	0	2 (6%)
not available	0	0	2 (11%)	0	2 (6%)
<10.000 copies/ml	4 (66%)	4 (44%)	8 (44%)	0	16 (47%)
≥10.000 copies/ml	1 (17%)	5 (56%)	7 (39%)	1	14 (41%)
not available	1 (17%)	0	3 (17%)	0	4 (12%)
<100.000 copies/ml	4 (66%)	6 (67%)	11 (61%)	0	21 (62%)
≥100.000 copies/ml	1 (17%)	3 (33%)	4 (22%)	1	9 (26%)
not available	1 (17%)	0	3 (17%)	0	4 (12%)
*Stage of disease*					
I	0	0	0	0	0
II	1 (17%)	0	1 (5%)	0	2 (6%)
III	1 (17%)	2 (22%)	5 (28%)	0	8 (24%)
IV/Burkitt leukemia	0	1 (11%)	5 (28%)	0	6 (18%)
not available	4 (66%)	6 (67%)	7 (39%)	1	18 (53%)
*Site of involvement*					
single site	0	0	1 (6%)	0	1 (3%)
graft	0	2 (22%)	2 (11%)	0	4 (12%)
lungs	0	1 (11%)	3 (17%)	0	4 (12%)
lymph nodes regarless of other sites	5 (83%)	9 (100%)	16 (89%)	1	31 (91%)
lymph nodes only	1 (17%)	3 (33%)	4 (22%)	0	8 (24%)
gastrointestinal tract	2 (33%)	2 (22%)	3 (17%)	0	7 (21%)
upper airway	3 (50%)	1 (11%)	6 (33%)	0	10 (29%)
central nervous system	0	0	2 (11%)	0	2 (6%)
bone marrow	0	1 (11%)	4 (22%)	0	5 (15%)
others	0	2 (22%)	6 (33%)	1	9 (27%)

Abbreviations: No., number; EBER, EBV‐encoded RNA; EBV, Epstein–Barr virus; PCR, polymerase chain reaction; PB, peripheral blood; PTLD, post‐transplant lymphoproliferative disease.

We aimed at staging the 34 patients according to the IPNHLSS. In 18 patients staging was incomplete, as pre‐therapeutic bone marrow (BM) and cerebrospinal fluid (CSF) were not assessed. Of the 16 patients evaluable for staging, two showed stage II disease, eight stage III disease, and six stage IV disease or Burkitt's leukemia. The patient with Burkitt's leukemia showed lesions in the CNS, too.

The most frequently involved sites of disease were the lymph nodes (n = 31/34), however, only in 8/34 patients the disease was solely restricted to the lymph nodes. Other sites of involvement were the gastrointestinal tract (GIT; n = 7/34), upper airways including the Waldeyer's ring (n = 10/34), lungs (n = 4/34), BM (n = 5/34), CNS (n = 2/34), and others (n = 9/34). Graft involvement was diagnosed in four cases (lungs, n = 1; kidney, n = 1; GIT, n = 2). Only 1/34 patients presented with uni‐locular disease (lingula).

Eighteen/34 cases were classified as monomorphic PTLD, all of which were of B‐cell origin. Diffuse large B‐cell lymphoma (DLBCL) accounted for the vast majority of this group (n = 15) occurring after SOT in 12/15 and after HSCT in 3/15 cases. The other three patients had a plasmacytoma‐like PTLD, plasmablastic lymphoma, and Burkitt's leukemia, respectively, all occurring after SOT. Polymorphic disease occurred in 9/34 cases, and non‐destructive lesions in 6/34 cases, all occurring after SOT. The latter group included five patients with plasmacytic hyperplasia (PH) and one with mononucleosis‐like PTLD. In 1/34 patients (after HSCT), the PTLD could not be subclassified. In two patients, synchronous detection of different subtypes was seen: one case of polymorphic disease showed elements of mononucleosis‐like PTLD, and one case with PH showed aspects of florid follicular hyperplasia. Twenty‐eight/34 demonstrated CD20‐expression. The CD20‐negative cases included plasmacytic hyperplasia (n = 2), polymorphic PTLD (n = 1), plasmablastic lymphoma (n = 1), and DLBCL (n = 2), respectively.

Depending on the latency period between transplantation and occurrence of PTLD, 19 occurred within the first year after transplantation. The median interval between transplantation and PTLD diagnosis was 0.65 years for all patients. All four cases of PTLD following HSCT occurred within the first year.

In 32/34 patients, serum PCR analysis was performed for EBV detection at the time of diagnosis with 30/32 being positive. EBV‐positive disease was defined by histological examination of the tumor, showing that 30 were EBER‐positive. Notably, two patients presented with EBER‐positivity of the samples, but had negative EBV‐PCR in PB.

All nine cases of polymorphic PTLD presented with EBV‐positive PTLD, while this was seen in 4/6 and 16/18 of non‐destructive and monomorphic PTLD, respectively. The patient with non‐classifiable PTLD was EBV‐positive. Of all cases occurring within 1 year after transplantation, all but one patient showed EBER‐positivity (n = 18/19). The remaining patient had a negative EBER‐status of the tumor, but positive EBV‐PCR in PB. Of the four EBV‐negative cases, two had non‐destructive and two monomorphic PTLD, and, interestingly, all were CD20‐negative. In three of the four cases with negative EBER‐reactions, EBV was detectable in PB.

### 4.2 Treatment and response

**FIGURE 1:**
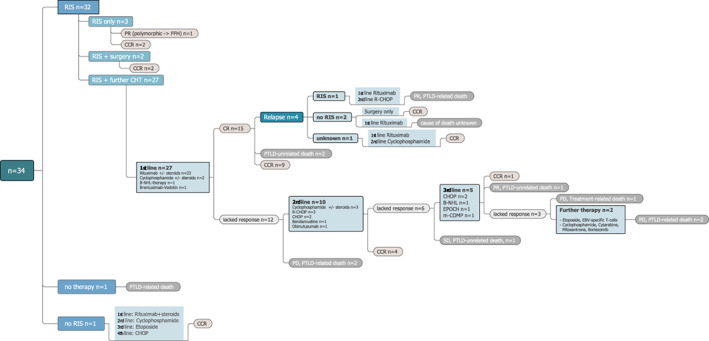
Flow chart of the therapy and outcome of the 34 PTLD patients. RIS, reduction of immunosuppression; PR, partial remission; FFH, florid follicular hyperplasia; CCR, continuous complete remission; B‐NHL, B‐cell non‐Hodgkin lymphoma; PTLD, post‐transplant lymphoproliferative disease; CHOP, cyclophosphamide, doxorubicin, vincristine and prednisolone; CR, complete response; R‐CHOP, rituximab‐CHOP; PD, progressive disease; EPOCH, etoposide, prednisolone, vincristine, cyclophosphamide and doxorubicin; m‐COMP, modified ‐ cyclophosphamide, vincristine, methotrexate and prednisone; SD, stable disease; EBV, Epstein–Barr virus

A detailed description of the therapy, response and outcome of the 34 patients is given in Figure 1 and Table 3. One/34 patients died from disease before any therapy. Reduction of immunosuppression (RIS) was the first therapeutic step taken in 32 of the remaining 33 individuals. The patient without RIS directly received rituximab followed by chemotherapy, which led to a CR. Three/32 patients with RIS required no further therapy. Two of them achieved a CR, the remaining patient's lesions switched from polymorphic to non‐destructive PTLD (classified as PR). Two/32 patients with RIS, including one with localized disease of the lingula and one with a supraglottic tumor and cervical lymphadenopathy, had a surgical approach of the disease only. Thereby both achieved a CR.

**TABLE 3:** Response and outcome of the 34 patients with PTLD according to the histological subtype

	Non‐destructive PTLD	Polymorphic PTLD	Monomorphic PTLD	Not classifiable PTLD	∑
*No. of patients*	6 (18%)	9 (27%)	18 (53%)	1 (3%)	34
*Response of initial disease*					
complete response	6 (100%)	5 (56%)	14 (78%)	0	25 (74%)
partial response	0	1 (11%)	1 (6%)	0	2 (6%)
stable disease	0	1 (11%)	0	0	1 (3%)
progressive disease	0	1 (11%)	3 (17%)	1	5 (15%)
not evaluable	0	1^a^ (11%)	0	0	1 (3%)
*First Event*					
no event	4 (67%)	3 (33%)	6 (33%)	0	13 (38%)
lack of complete response	2 (33%)	3 (33%)	8 (44%)	1	14 (41%)
relapse	0	2 (22%)	2 (11%)	0	4 (12%)
death	0	1	2	0	3 (9%)
*Response of first relapse*	0	2	2	0	4
complete response	0	1 (50%)	1 (50%)	0	2 (50%)
partial response	0	1 (50%)	0	0	1 (25%)
stable disease	0	0	0	0	0
progressive disease	0	0	0	0	0
unknown	0	0	1 (50%)	0	1 (25%)
*Overall outcome*					
median follow‐up (years)	1.27	7.81	6.48	/	5.68
range of follow‐up (years)	0.37‐2.54	1.45‐16.05	0.14‐19.56	/	0.14‐19.56
CCR	6 (100%)	4 (44%)	11 (61%)	0	21 (62%)
First CCR	6 (100%)	3 (33%)	10 (56%)	0	19 (56%)
Second CCR	0	1 (11%)	1 (6%)	0	2 (6%)
death	0	4 (44%)	7 (39%)	1	12 (35%)
PTLD‐related	0	3 (33%)	3 (17%)	0	6 (18%)
therapy of PTLD‐related	0	0	0	1	1 (3%)
not‐PTLD‐related	0	1 (11%)	3 (17%)	0	4 (12%)
cause of death unknown	0	0	1 (6%)	0	1 (3%)

Abbreviations: No., number; CCR, continuous complete remission; PTLD, post‐transplant lymphoproliferative disease.

^a^ One patient did not receive any therapy at all.

Of the 27/32 patients with RIS who received further treatment, 15 achieved a CR, while 12 lacked complete response including two progressing during first‐line therapy and dying from the PTLD. Four/15 patients with CR following first‐line therapy relapsed, 2/15 died in first CR due to PTLD‐unrelated causes and 9/15 remained in first continuous CR (CCR). Two of the four relapsed patients achieved a second CCR, the other two died (unknown cause, n = 1; PTLD‐related, n = 1).

Out of the 12 patients who lacked complete response to first‐line therapy, four achieved a first CCR with second‐line therapy, one with stable disease died from a PTLD‐unrelated cause, and five lacked complete response to second‐line therapy. One of the latter achieved a first CCR following third‐line therapy, one attained a PR and died (PTLD‐unrelated), and another patient progressed and died from third‐line therapy‐associated toxicity. The remaining two patients lacking complete response to third‐line therapy proceeded to further chemotherapy, but subsequently succumbed to PTLD.

### 4.3 Outcome according to therapy

Twenty‐three/27 patients with RIS and further therapy were treated with rituximab as part of their first‐line therapy with or without additional steroids. First‐line therapy in the remaining four consisted of cyclophosphamide and steroids in two, and brentuximab‐vedotin and B‐cell non‐Hodgkin lymphoma (B‐NHL)‐based chemotherapy in each one patient. Fifteen of the 27 patients achieved a CR.

Ten/12 patients with RIS and additional treatment, who lacked complete response to first‐line therapy, proceeded to polychemotherapy in nine (R‐CHOP, n = 3; CHOP, n = 2; cyclophosphamide±steroids, n = 3; bendamustine, n = 1) and obinutuzumab in one patient. Five/six patients without complete response to second‐line therapy proceeded to chemotherapy (CHOP, n = 2; B‐NHL therapy, n = 1; EPOCH, n = 1; m‐COMP, n = 1), and two of three patients, who lacked complete response to third‐line therapy, subsequently received other therapies such as etoposide and EBV‐specific T‐cells in one, and cyclophosphamide followed by cytarabine, mitoxantrone, and bortezomib in the other patient.

Of all 14 patients treated with rituximab only (in addition to RIS), 13 achieved a CR, one showed disease progression at the end of therapy, and three patients relapsed. Classical B‐NHL therapies were applied in one case of Burkitt's leukemia as first‐line and in another case of DLBCL as third‐line therapy. The latter achieved a PR and died from a PTLD‐unrelated cause; the former achieved a CCR.

### 4.4 Outcome according to histology

Of all six patients with non‐destructive disease, four received rituximab±steroids, and two lacked complete response and received second‐line therapy (Table 3). All patients finally achieved a CCR.

Among the nine patients with polymorphic disease, eight received RIS upfront, and one patient died before having received any therapy. Two/eight patients received RIS only, by which one achieved a CCR. The other patient's lesions regressed from polymorphic to non‐destructive PTLD (classified as PR). Six/8 patients received rituximab±steroids, with four achieving a CR and two who did not (both died). Two of the four patients relapsed, of whom one died from the PTLD, and the other one achieved a second CCR. Of all eight patients with polymorphic disease having received treatment, three achieved a first CCR, and one a second CCR, while one showed a PR and three patients died (PTLD‐related, n = 2; PTLD‐unrelated, n = 1) (Table 3).

Monomorphic PTLD was documented in 18 cases, including the only patient without RIS upfront. One/18 achieved a CCR by RIS and surgery, the other 17 received additional therapy (rituximab±steroids, n = 13; cyclophosphamide±steroids, n = 2; B‐NHL therapy, n = 1; brentuximab‐vedotin, n = 1). Eleven/17 patients received first‐line therapy only, whereby nine achieved a CR, and the other two died of progressive disease. Of the nine patients in CR after first‐line treatment, five remained in CCR, two relapsed (death, n = 1; second CCR, n = 1), and two died from a PTLD‐unrelated cause.

Six/17 patients received second‐line therapy (cyclophosphamide, n = 2; R‐CHOP, n = 2; CHOP, n = 1; bendamustine, n = 1), by which two achieved a CR, whereas four proceeded to third‐line therapy due to lack of complete response (B‐NHL therapy, n = 1; etoposide, n = 1; m‐COMP, n = 1; EPOCH, n = 1). Thereby, one achieved a CCR, and another one died from a PTLD‐unrelated cause. The remaining two patients subsequently received further therapy, by which one patient achieved a CCR, and the other one died from PTLD progression. Overall, 11/18 patients with monomorphic disease achieved a CCR, while 7/18 patients died (PTLD‐related, n = 3, PTLD‐unrelated, n = 3, unknown cause, n = 1).

The one/34 patients with unclassifiable PTLD received RIS and rituximab+steroids first, to which he lacked response and proceeded to cyclophosphamide and polychemotherapy (CHOP). He died from a treatment‐related complication.

Notably, two/24 patients having received rituximab within first‐line therapy showed CD20‐negative disease. One of them presented with plasmacytic hyperplasia and was treated with RIS + rituximab, and the other one had a DLBCL and received rituximab followed by chemotherapy (cyclophosphamide, etoposide, and CHOP). Both achieved a CR.

### 4.5 Outcome according to type of transplantation

Response and outcome of the 4 HSCT as compared to the 30 SOT patients are shown in Supplemental Table 1. Although numbers are small, results suggest a worse response and outcome for the HSCT patients.

### 4.6 Events

Thirteen/34 patients experienced no events and are alive in first CCR. Fourteen/34 patients lacked complete response to first‐line therapy. Out of them, seven died (PTLD‐related, n = 4, PTLD‐unrelated, n = 2, treatment‐related, n = 1). Four/34 patients relapsed, of whom 2 died (PTLD‐related, n = 1; unknown cause, n = 1). Among the remaining 3/34 patients, death occurred as a first event, of which one was PTLD‐related, and the other two were PTLD‐unrelated.

Six/12 deaths were considered PTLD‐related, with five occurring during first‐line treatment (progression, n = 4; EBV‐associated hemophagocytic lymphohistiocytosis, n = 1). The remaining one died from PTLD‐related gastrointestinal perforation during relapse therapy. One patient died amidst relapse therapy due to an unknown cause. One case of candida septicemia was considered a PTLD‐treatment‐related death. The group of 4/12 PTLD‐unrelated deaths consisted of a septicemia, an acute respiratory distress syndrome combined with secondary graft failure following HSCT, a diffuse alveolar hemorrhage syndrome, and a cardiac arrest, respectively.

### 4.7 Survival rates

**FIGURE 2:**
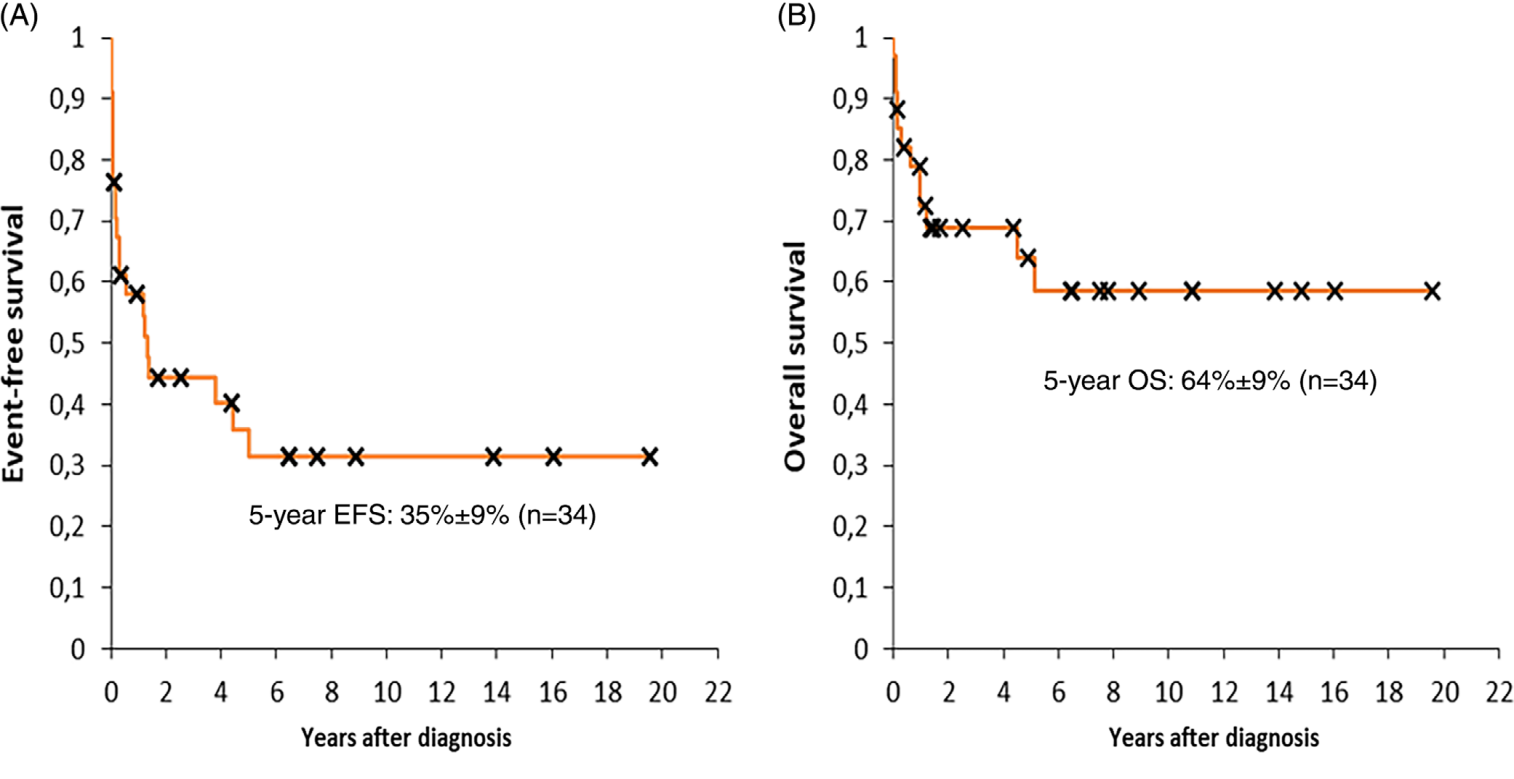
5‐year event‐free survival (A) and overall survival (B) of the 34 patients with PTLD. EFS, event‐free survival; OS, overall survival. [Correction added on 12 April 2021, after first online publication: The text “5‐year EFS: 35%±9% (n=34)” has been added in figure 2 A image in this version]

Median follow‐up for surviving patients was 5.68 years as calculated from the time of diagnosis of primary PTLD diagnosis. On the date of last follow‐up 22 of all 34 patients were alive in CCR. The 5‐year OS and EFS rates were 64% ± 9% and 35% ± 9% for the whole study cohort, respectively (Figure 2). When evaluating the 5‐year OS according to the PTLD subtypes, it was 100% for non‐destructive, 53% ± 17% for polymorphic, and 65% ± 12% for monomorphic PTLD. Five‐year EFS according to the PTLD subtypes was 53% ± 25% for non‐destructive, 33% ± 16% for polymorphic, and 36% ± 12% for monomorphic PTLD.

A variety of factors were evaluated concerning their impact on OS and EFS (Supplemental Table 2): Diagnosis indicating transplantation was the only factor having an effect on OS with a significantly poorer survival in cases with malignant diseases (*P* = 0.002), though numbers were very small. None of the factors analyzed had a significant influence on EFS.

## 5 DISCUSSION

PTLD represents a heterogenous group of excessive lymphoid proliferation, generally B‐lymphocytes, that occurs in the setting of suppressed T‐cell function, usually after SOT. In HSCT patients, it often represents a fatal risk occurring relatively early after transplantation. EBV infection has long been implicated as a causative factor in the development of PTLD.^8^, ^11^, ^21^, ^45^, ^46^, ^47^, ^48^ In our study, 30 of 34 cases showed a positive EBER‐reaction of the tumor, thereby confirming the high rates of association seen in previous pediatric studies. Our data matched previous reports on monomorphic and polymorphic PTLD with 16/18 and 9/9 of the patients being EBER‐positive, while only 4/6 patients with non‐destructive PTLD were EBER‐positive. Interestingly, among the four EBV‐negative cases, median time from transplantation until PTLD was 6.0 years as compared to 2.3 years among the EBV‐positive patients. Accordingly, all four EBER‐negative patients had a CD20‐negative tumor, but no reduced survival.

The incidence of LPD following transplantations shows a biphasic distribution, with a first peak occurring within 12 months, and another either at 3 to 5 or at 7 to 10 years after transplantation.^11^, ^25^, ^49^, ^50^, ^51^, ^52^ Our study showed that half of the patients presented with early‐onset disease at a median time of 0.44 years and half with late‐onset disease at a median time of 5.61 years after transplantation. In accordance with the literature, all four patients with PTLD after HSCT were diagnosed within the first year after transplantation.^9^, ^37^, ^53^

The majority of PTLDs present as multi‐locular disease, affecting both nodal and extranodal sites. Virtually every organ can be involved, the graft itself being a common site, whereas the most frequently affected extranodal site is the intestine.^15^, ^54^, ^55^, ^56^ We found that the vast majority (n = 33) involved multiple sites and the most frequently affected site were the lymph nodes (n = 31) followed by the upper airways (n = 10) and intestinal tract (n = 7). The graft itself was affected in four of our 34 cases.

Since experience with PTLD in childhood is limited to small case series and studies, treatment is not fully standardized and frequently adjusted to the affected individual, thereby addressing the multitude of clinical and histological presentations. Restoration of the patients' EBV‐specific T‐cell response achieved by RIS is widely accepted as the treatment of first choice independent of the organ graft. However, there is no consensus about the impact of a single immunosuppressive agent and the risk of acute graft‐rejection is assessed differently by the respective organ specialist.^11^, ^35^, ^57^, ^58^ Remarkably, none of the 32 patients included in our study who received RIS suffered from graft‐failure or ‐loss due to PTLD‐therapy. Non‐destructive PTLD and low‐risk patients show high response rates to RIS alone, but it is not recommended as a sole therapy in cases of monomorphic PTLD, aggressive disease or patients with a high tumor burden.^11^, ^35^ Among our cohort, three underwent RIS only (non‐destructive, n = 1; polymorphic, n = 2) and thereby achieved a CCR (n = 2) or PR (n = 1), and another 2 patients achieved CCR by RIS and surgery only (non‐destructive, n = 1; monomorphic, n = 1).

Another substantial part of PTLD‐therapy is B‐cell depletion due to the use of rituximab. It is recommended as first‐line treatment for all CD20‐positive subtypes sequential or in parallel to RIS. Altogether, treatment with rituximab has led to better overall survival in children and adolescents with PTLD.^22^, ^59^, ^60^, ^61^ Eighty‐five percent of our patients having received first‐line treatment required further therapy, with 86% of them having received rituximab±steroids as first‐line therapy. Forty‐six percent of patients lacked response to rituximab, including one patient who died from progressive disease and all others proceeding to chemotherapy. Of the 54% of patients having achieved CR after rituximab‐containing therapy, 23% relapsed.

In cases of DLBCL non‐responding to RIS and rituximab, as well as “non‐DLBCL” monomorphic PTLD and primary CNS‐lymphoma, polychemotherapy is suggested, mostly consisting of R‐CHOP, CHOP, and COP regimens.^35^, ^62^ Overall, 56% of our patients with monomorphic disease underwent polychemotherapy at some time, 40% of whom already received it as first‐line therapy, whereas 60% proceeded to chemotherapy after lacking response to rituximab±steroids or brentuximab‐vedotin.

Novel therapeutic approaches include immunotherapies, such as EBV‐specific CTLs and chimeric antigen receptor T‐cells, small molecule inhibitors, such as the mTOR inhibitor everolimus or tyrosine kinase inhibitor ibrutinib, as well as risk‐adapted chemotherapy regimens. Aside from rituximab, targeted therapies such as the anti‐CD30 antibody brentuximab‐vedotin yield promising results.^11^, ^35^, ^63^ Within our cohort, one patient with plasmablastic lymphoma received brentuximab‐vedotin as first‐line therapy, but proceeded to further chemotherapy due to refractory disease.

Several factors reportedly influence the prognosis of pediatric patients with PTLD.^13^, ^36^, ^60^, ^64^, ^65^, ^66^, ^67^, ^68^, ^69^, ^70^, ^71^, ^72^, ^73^ Parameters associated with a poor prognosis are a history of malignant disease indicating transplantation, advanced disease, multifocal and extranodal disease, CNS, BM and graft involvement, female gender, B‐symptoms and elevated LDH levels at PTLD onset, high EBV load in PB at the time of diagnosis, CD20‐ and EBV‐negative as well as monomorphic and late‐onset PTLD. Considering the type of allograft, lung, liver, and hematopoietic stem cells were associated with poorer prognosis. None of the factors referred to could be identified as relevant prognostic factors affecting EFS or OS in our patient cohort, which may at least be partially owed to the relatively small number of patients included in our study.

Children diagnosed with PTLD tend to have a better prognosis compared to adults, which seems to be due to more favorable PTLD‐subtypes (EBV‐positive) and less treatment‐related complications.^15^, ^20^ The prospective PTLD‐1 trial implemented sequential treatment with rituximab followed by a CHOP‐regimen in adult CD20‐positive patients with mostly monomorphic PTLD, resulting in a median OS of 6.6 years. Considering that response to rituximab predicted favorable OS, another prospective study subsequently treated the patients with CR to 4 weeks of rituximab with further rituximab consolidation, whereas non‐responders switched to R‐CHOP. Thereby, a 3‐year OS of 78% (compared to 69% in PTLD‐1) was achieved.^59^, ^61^ The prospective Ped‐PTLD 2005 trial treated pediatric patients with CD20‐positive PTLD following SOT with 3 weeks of rituximab followed by either rituximab or chemotherapy (mCOMP) depending on initial response, achieving a 2‐year OS of 86%, and 67% surviving event‐free.^64^ According to the literature available, 5‐year survival rates for pediatric PTLD are around 53‐80%.^25^, ^61^, ^65^, ^74^, ^75^ The 3‐ and 5‐year OS rates in our patient cohort were 69% ± 8% and 64% ± 9%, respectively, fitting well to the hitherto reports.

The primary aim of this study was to investigate the characteristics and outcome of PTLD in Austrian patients over a period of 20 years. Special attention was paid to factors influencing the patient's prognosis. In childhood and adolescence, PTLD is the largest group of secondary malignant diseases, making it essential to define coherent diagnostic and therapeutic guidelines addressing the heterogeneity of this disease. Considering the absolute number of affected people, it is still defined as an Orphan Disease, and both, a transnational cooperation and a compilation of comprehensive scientific studies, including series like the present one, are essential to optimize the management of PTLD, as it is still a major cause of mortality following transplantation.

## CONFLICT OF INTEREST

The authors declare no competing financial interests.

## AUTHORS' CONTRIBUTIONS

A.A., A.F., and R.C. designed and planned the study; A.A., A.F., G.K., and R.C. wrote the manuscript; A.A., A.F., and G.K. collected and analyzed the data; I.S.K. was in charge of the histopathological analyses; M.B., M.D., S.G., W.D.H., H.H., A.L., G.M., I.M.B., T.M.S., H.P., W.S., Z.S., A.A., G.K., and R.C. recruited, treated, and identified the patients. A.F., G.K., M.B., M.D., S.G., W.D.H., H.H., A.L., G.M., I.M.B., T.M.S., H.P., I.S.K., W.S., Z.S., R.C., and A.A. read and approved the final version of the manuscript.

## ETHICAL STATEMENT

Ethical approval for this study (No. 1919/2018) was granted by the Ethics Committee of the Medical University of Vienna on 11th November 2018.

## PATIENT CONSENT STATEMENT

This was a retrospective study involving access to existing medical records, with the research personnel reviewing these records normally having access to these records. Therefore, patient consent was waivered.

## Supporting information


**Supplemental Table 1** Response and outcome of the 4 HSCT patients as compared to the 30 SOT patients with PTLD

**Supplemental Table 2** Event‐free and overall survival of the 34 patients with PTLD according to prognostic factors analyzed

## Data Availability

The data that support the findings of the study are available on request from the corresponding authors.
