# Supplementary material for: Characteristics, management, and outcome of pediatric patients with post‐transplant lymphoproliferative disease—A 20 years' experience from Austria
Source: Cancer Rep (Hoboken). 2021 Mar 23;4(5):e1375. doi: 10.1002/cnr2.1375 (PMC8551996; doi:10.1002/cnr2.1375)
Supplement: Supplementary file 1 — Supplemental Table 1 Response and outcome of the 4 HSCT patients as compared to the 30 SOT patients with PTLD [file CNR2-4-e1375-s001.docx]

**Supplemental Table 1. Response and outcome of the 4 HSCT patients as compared to the 30 SOT patients with PTLD**

**Abbreviations:** No., number; CCR, continuous complete remission; PTLD, post-transplant lymphoproliferative disease; HSCT, hematopoietic stem cell transplantation; SOT, solid organ transplantation.

* 1 patient did not receive any therapy at all.
