# Supplementary material for: Characteristics, management, and outcome of pediatric patients with post‐transplant lymphoproliferative disease—A 20 years' experience from Austria
Source: Cancer Rep (Hoboken). 2021 Mar 23;4(5):e1375. doi: 10.1002/cnr2.1375 (PMC8551996; doi:10.1002/cnr2.1375)
Supplement: Supplementary file 2 — Supplemental Table 2 Event‐free and overall survival of the 34 patients with PTLD according to prognostic factors analyzed [file CNR2-4-e1375-s002.docx]

**Supplemental Table 2. Event-free and overall survival of the 34 patients with PTLD according to prognostic factors analyzed**

**Abbreviations:** Tx, transplantation; PTLD, post-transplant lymphoproliferative disease; EBER, EBV-encoded RNA; EBV, Epstein-Barr virus; PCR, polymerase chain reaction; PB, peripheral blood; LDH, lactate dehydrogenase; No., number; EFS, event-free survival; OS, overall survival; BM, bone marrow; CNS, central nervous system; n.s., not significant.

* in 4 patients the EBV-PCR load in PB was not known.

# 1 patient did not receive any therapy at all.
